# Hyperglycemia effect on coronary disease in patients with metabolic syndrome evaluated by intracoronary ultrasonography

**DOI:** 10.1371/journal.pone.0171733

**Published:** 2017-02-10

**Authors:** Beatriz Dal Santo Francisco Bonamichi, Erika Bezerra Parente, Ana Carolina Noronha Campos, Adriano Namo Cury, João Eduardo Nunes Salles

**Affiliations:** 1 Santa Casa de São Paulo Hospital, Internal Medicine Department, Endocrinology Unit, São Paulo, Brasil; 2 Santa Casa de São Paulo Hospital, Internal Medicine Department, São Paulo, Brasil; Universitatsklinikum Freiburg, GERMANY

## Abstract

**Introduction:**

Metabolic syndrome (MS) is characterized by dyslipidemia, central obesity, hypertension and hyperglycemia. However, type 2 diabetes mellitus (T2DM) may or may not be present in metabolic syndrome. MS and T2DM are considered important cardiovascular risk factors, but the role of hyperglycemia in coronary disease is still contested in the literature. Therefore, we decided to evaluate the effect of hyperglycemia on the severity of coronary disease in MS patients, with or without T2DM, submitted to coronary angiography (CA) and intravascular ultrasonography (IVUS).

**Materials and methods:**

This is a cross sectional, observational study with 100 MS patients (50% with T2DM), 60% male. All of the patients had been referred for CA procedures. The obstruction was considered severe when stenosis was greater than 70% and moderate if it was between 50–69%. Patients detected with a moderate obstruction by CA were indicated to IVUS. A minimal luminal area of less than 4mm^2^ detected by IVUS was also considered severe. IDF criteria were used to define Metabolic Syndrome and T2DM diagnosis was defined according to the American Diabetes Association criteria. Student’s t-test and Pearson Chi-square were used for statistical analysis, considering p < 0.05 statistically significant.

**Results and discussion:**

The majority of T2DM patients presented severe arterial lesions (74% vs 22%, p<0.001). Using CA procedure, 12% of T2DM had moderate obstructions, compared to 38% of the non-diabetic group (p< 0.05). 8% of patients with moderate lesions by CA were diagnosed with a luminal area less than 4mm^2^ using IVUS. This luminal area was significantly smaller in the T2DM group than in the control group (3.8mm^2^ ± 2.42. vs 4.6mm^2^ ± 2.58, p = 0.03).

**Conclusion:**

Patients with MS and T2DM submitted to angiography and IVUS, had more severe coronary lesions compared to MS patients without diabetes. This finding suggests that beyond insulin resistance that is present in MS, hyperglycemia may also play a role in the development of atherosclerotic disease. IVUS was useful for diagnosing 8% of severe cases initially considered to be moderate obstructions when using just CA in this scenario.

## Introduction

Insulin resistance with dyslipidemia, central obesity, hypertension and hyperglycemia characterize Metabolic Syndrome (MS)[1]. The prevalence of MS in the United States is about 20–25% and it is considered as important cardiovascular (CV) risk factor as smoking[2]. Type 2 diabetes mellitus (T2DM) is also a major CV risk factor, which may or may not be present in MS. The role of glycemia in macro vascular disease however, is still contested[3].

Atherosclerosis is an insidious disease and it is possibly the most important risk factor for the development of cardiovascular events. Some studies have suggested that the progression to T2DM from insulin resistance status occurs in parallel with the progression of atherosclerosis from endothelial dysfunction[4]. The United Kingdom Prospective Diabetes Study (UKPDS) showed fewer CV events after ten years of good glycemic control[5]. However, other studies, such as ACCORD, ADVANCE and VADT did not[6–8]. The question that has remained unanswered until now is: what is the real role of hyperglycemia in the pathogenesis of cardiovascular disease? In addition, if hyperglycemia is really an important factor in developing atherosclerosis and cardiovascular events, what is the relevance of glycemic control to avoid these complications?

In our study, we decided to evaluate the severity of coronary atherosclerotic disease in patients with metabolic syndrome, with or without diabetes, to better understand the role of hyperglycemia in the process of atherosclerosis. The primary objective of this study is to evaluate the influence of hyperglycemia on the severity of coronary atherosclerotic disease in patients with MS with or without T2DM, submitted to coronary angiography (CA). The secondary objective is to evaluate the percentage of false negatives for severe coronary disease among patients classified as having moderate obstructions using CA, when submitted to intravascular ultrasonography (IVUS).

## Materials and methods

This study was approved by Santa Casa Ethics Committee according to the principles expressed in the Declaration of Helsinki. All patients signed the written informed consent and all these documents are stored at the research center.

It is an observational, cross sectional, controlled study, with 100 patients with metabolic syndrome, 50% of whom had T2DM. Data were collected from Santa Casa de São Paulo Hospital using cardiology outpatient and inpatient charts, between March and December, 2014. We considered as inclusion criteria patients with metabolic syndrome referred for CA procedures to investigate coronary disease. A coronary obstruction was considered severe when stenosis was greater than 70% and moderate if it was between 50–69%[9]. Patients detected with a moderate obstruction by angiography were indicated to IVUS. A minimal luminal area found using IVUS was considered severe if it was less than 4 mm^2^[10]. We measured glycemia, lipids, body weight, abdominal circumference and blood pressure to define metabolic syndrome. IDF criteria were used to define Metabolic Syndrome[11] and T2DM diagnosis was defined according to the American Diabetes Association criteria[12].

The same physician conducted all CA and IVUS procedures. IVUS imaging were performed according to recommended standards, using the Atlantis SR Pro Imaging Catheter 40 MHz connected to the iLab Ultrasound Imaging System (both by Boston Scientific Corporation, Natick, Massachusetts). The vessels were imaged during automated pullback at 0.5 mm/s, but additional manual runs were strongly encouraged to allow for detailed analysis of specific issues. An online IVUS Radiofrequency (IVUS–RF) backscatter analysis technology was used to assess the minimum luminal area. We recorded these components in absolute terms and volume plaque area.

Santa Casa Hospital laboratory analyzed blood samples (glycemia, glycated hemoglobin, cholesterol and triglycerides levels). We used Student’s t-test and Pearson Chi-square for statistical analysis, considering p < 0.05 as statistically significant.

## Results

There was no statistically significant difference between the two groups at the beginning of the study, exception for the fasting glucose and glycated hemoglobin, the variables necessary to differentiate the patients with and without diabetes (Table 1). All patients had systemic arterial hypertension. There were no smokers in either group and the proportion of men and women in each group was similar (30 men and 20 women). The patients in the diabetic group had been diagnosed 8 ± 2 years before. The mean age was 61 ± 5 years for the T2DM group and 63 ± 4 years for the non-T2DM group.

**Table 1:** Baseline characteristics.

	T2DM	Not T2DM	P-value
SBP mmHg	135±4	138 ± 3	0.12
DBP mmHg	89 ± 2	85 ± 4	0.09
WC cm	109±5	106 ± 4	0.08
Glycemia mg/dl	131±6	93 ± 5	0.02
HbA1c mg/dl	7.7±1.4	5.8±0.4	0.03
TC mg/dl	195±9	198 ± 6	0.09
HDL-C mg/dl	49 ± 4	52 ± 5	0.08
LDL-C mg/dl	128±8	135 ±4	0.11
Tg mg/dl	181±5	175 ±7	0.06

SBP: systolic blood pressure, DBP: diastolic blood pressure, WC: waist circumference, HbA1c: glycated hemoglobin, Tg: triglycerides, TC: total cholesterol

**Fig 1:**
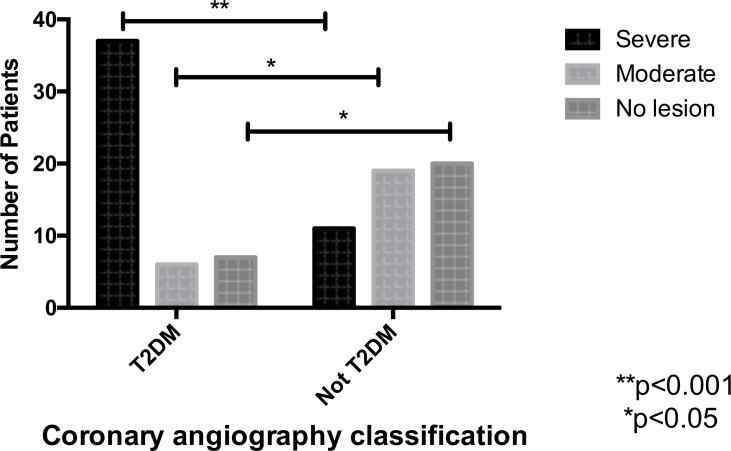
Coronary angiography classification. *p< 0.05 **p< 0.001.

**Fig 2:**
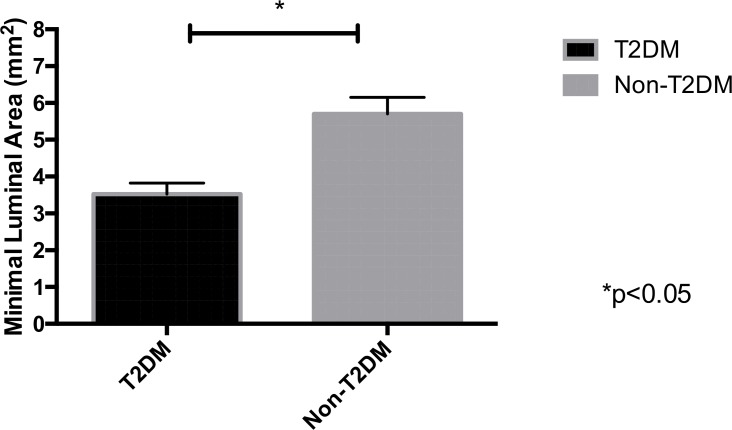
Minimal luminal area. *p< 0.05.

In the diabetic group, 74% (37) of patients had severe coronary lesions and 12% (6) of them had moderate obstructions, compared to 22% (11) with severe and 38% (19) with moderate obstructions in the non-diabetic group (Fig 1). All 25 patients with moderate lesions (25% of the original study population) screened using CA were indicated to IVUS. After IVUS, 8% of these were diagnosed with severe coronary obstructions with luminal area less than 4 mm^2^. The minimal luminal area was smaller in the diabetic group than in the control group (3.6mm^2^ ± 0.23. vs 4.7mm^2^ ± 0.31, p = 0.03). (Fig 2).

## Discussion

Coronary disease is a high morbidity and mortality disease especially in people with T2DM[13]. Studies have shown that T2DM patients have 3 to 4 times more risk of a coronary event than non-diabetic patients[2,13,14]. Nevertheless, the real role of hyperglycemia in the pathogenesis of atherosclerosis disease leading to a CV event is still unknown.

In our study, we found that people with MS and hyperglycemia (T2DM) had more severe coronary lesions than people with the same metabolic risk factors like dyslipidemia, increased abdominal circumference and high blood pressure, but no diabetes. This finding suggests that beyond insulin resistance that is present in MS and also in T2DM, hyperglycemia itself may have an impact on the pathogenesis of atherosclerosis disease. These findings agree with other studies that demonstrated a parallel between T2DM progression from insulin resistant status and the progression of atherosclerosis from endothelial dysfunction[15–17].

In another article, using a similar approach to assess hyperglycemia and coronary disease, Yang et al. showed using IVUS that patients with T2DM and good glycemic control had better coronary plaque composition and smaller plaque volume than patients with diabetes and no glycemic control. The authors suggested that hyperglycemia control is important in the progress of cardiovascular disease[18]. Although our study focus was not on plaque composition, but on obstruction coronary artery level and minimum luminal area, we also could demonstrate that in patients with coronary disease (all 100 patients had coronary lesions), MS and hyperglycemia exhibited higher number of severe obstruction and smaller luminal minimum area than the other compared group of patients without hyperglycemia[18].

If we look at clinical data from large clinical trials, as UKPDS [5], we have seen after ten years of follow up that patients who had good glycemic levels from the outset had fewer CV events than those lacking controlled levels. Conversely, trials conducted by ACCORD[6], VADT[8] and ADVANCE[7] showed that glycemic control had no benefit in terms of CV endpoints. Nevertheless, there are some differences between the patients sampled in these studies, such as the number of years of diabetes diagnosis and the severity of diabetes complications, especially macrovascular ones. This variation suggests that timing of glycemic control is a critical factor in the development of CV disease[19]. However, after coronary obstruction disease establishment, glycemia probably does not make such difference. All of our patients had coronary disease but no myocardial infarctions. They all had atherosclerosis disease and were probably progressing towards having a CV event. The only different risk factor was glycemia. As we found a smaller luminal area and a greater number of diabetic patients with severe coronary obstructions, we can suppose that hyperglycemia may play a role in the progression of atherosclerosis lesions. This is in agreement with the literature that demonstrated that people with diabetes have worse atheroma plaque composition with more inflammation, compared to people without diabetes[20].

Performing routine exams for coronary disease screening in cardiovascular asymptomatic patients was not been beneficial in terms of major CV events[21]. Nevertheless, our patients were symptomatic and after CA indication we found patients (25% of the total) with moderate lesions. When a patient is diagnosed with severe or no coronary obstruction, the treatment is well established, but for moderate lesions it is still controversial. With IVUS it is possible to obtain data regarding the vessel's wall[22] and coronary minimal luminal area, that is the reason we decided to perform IVUS screening on our patients to investigate if an angiographically moderate lesion was severe or not[23]. With this new tool we found 8% of moderate lesions were severe, which requires a treatment because of the high risk of CV event. Intravascular ultrasound can also be used to determine the major predictors of restenosis and stent thrombosis, which are the main pitfalls of percutaneous coronary intervention [22]. Studies have shown that some coronary lesions considered moderate under CA screening are actually found to be severe when using IVUS, a finding that can affect the patient’s treatment[22–25]. According to the Brazilian Cardiology Society[26], a minimal coronary luminal area less than 4mm^2^ is indicative of the need for invasive treatment because of the high probability of a myocardial ischemia. Our study found severe obstructions in 8% of all patients who had moderate lesions, observed using CA. Since severe coronary disease raises the probability of developing a CV event, and this event has a high morbidity and mortality rate, we may consider that IVUS could be a useful tool to investigate coronary moderate lesions (shown by CA) in patients with metabolic syndrome.

## Limitations

Study limitations include its observational design and the small sample size.

## Conclusion

Our study showed that patients with MS and T2DM submitted to angiography were often found to have more severe coronary lesions and obstructions (less than 4mm^2^ minimal luminal area by IVUS), compared to patients with metabolic syndrome without diabetes. This result suggests that beyond insulin resistance, hyperglycemia may also play a role in the development of atherosclerotic disease. Another important result was that IVUS was a useful tool after angiography for diagnosing patients with severe disease which were misdiagnosed with moderate coronary lesion by CA.

## Supporting information

S1 Table A1c and minimal luminal area data. (XLSX)

## Abbreviations

MS: Metabolic Syndrome
CV: cardiovascular
T2DM: type 2 Diabetes Mellitus
CA: coronary angiography
IVUS: intravascular ultrasonography
SBP: systolic blood pressure
DBP: diastolic blood pressure
WC: waist circumference
Tg: triglycerides
TC: total cholesterol
HDL-C: high density level-cholesterol
LDL-C: low density level-cholesterol
